# The Whole-Body MRI Reporting and Data System Guidelines for Prostate Cancer (MET-RADS-P), Multiple Myeloma (MY-RADS), and Cancer Screening (ONCO-RADS)

**DOI:** 10.3390/cancers17020275

**Published:** 2025-01-16

**Authors:** Marco Parillo, Carlo Augusto Mallio

**Affiliations:** 1 Radiology, Multizonal Unit of Rovereto and Arco, APSS Provincia Autonoma Di Trento, 38123 Trento, Italy; 2 Fondazione Policlinico Universitario Campus Bio-Medico, 00128 Roma, Italy; 3 Research Unit of Diagnostic Imaging and Interventional Radiology, Department of Medicine and Surgery, Università Campus Bio-Medico di Roma, 00128 Roma, Italy

**Keywords:** whole-body magnetic resonance imaging, MRI scans, radiology, RADS, MET-RADS-P, MY-RADS, ONCO-RADS, practice guidelines, clinical oncology, narrative review

## Abstract

Whole-body magnetic resonance imaging (WB-MRI) is being employed with increasing frequency to evaluate a broader spectrum of patients with diverse types of cancer and for cancer screening purposes. While clinical guidelines support its use, a standardized radiological approach is still lacking. To improve consistency in the acquisition, interpretation, and reporting of WB-MRI examinations, three reporting and data systems (RADSs) have been recently suggested: METastasis Reporting and Data System for Prostate Cancer (MET-RADS-P), Myeloma Response Assessment and Diagnosis System (MY-RADS), and Oncologically Relevant Findings Reporting and Data System (ONCO-RADS). MET-RADS-P was developed to stage and monitor men with advanced prostate cancer using WB-MRI. It has emerged as a reliable imaging biomarker for predicting metastatic disease progression and assessing treatment response. MY-RADS was developed to stage and monitor patients with multiple myeloma using WB-MRI, emerging as a prognostic imaging biomarker. However, the evidence regarding inter-reader agreement for MY-RADS is currently limited. ONCO-RADS was developed to standardize the use of WB-MRI for cancer screening in individuals with cancer predisposition syndromes and in the general population. While initial findings are promising, the evidence supporting its use remains limited. To further validate and expand upon these promising preliminary findings, additional large-scale, prospective, multicenter studies are necessary.

## Simple Summary

Whole-body magnetic resonance imaging (WB-MRI) is increasingly being used to evaluate a wider range of patients with different types of cancer and for cancer screening. To standardize acquisition, interpretation, and reporting, three WB-MRI reporting and data systems (RADSs) have been recently introduced: METastasis Reporting and Data System for Prostate Cancer (MET-RADS-P), Myeloma Response Assessment and Diagnosis System (MY-RADS), and Oncologically Relevant Findings Reporting and Data System (ONCO-RADS). The aim of this review is to provide a summary of the scientific evidence that has emerged so far regarding the clinical utility of WB-MRI RADSs. Although preliminary studies have demonstrated promising and consistent results regarding the prognostic value and reliability of WB-MRI RADSs, additional research is necessary, particularly for ONCO-RADS and inter-reader agreement for MY-RADS. Larger, prospective, multi-center studies are required to confirm and extend these encouraging initial findings.

## 1. Introduction

Whole-body magnetic resonance imaging (WB-MRI) has been used in oncological imaging for over two decades, with particular interest thanks to its high diagnostic accuracy in identifying malignant disease across various organs and its ability to monitor tumor progression without ionizing radiation exposure [1]. Early studies demonstrated the utility of coronal short tau inversion recovery images, covering the entire body from head to pelvis, in detecting bone metastases in breast cancer patients within approximately 45 min [2,3]. Recent technological advancements have significantly improved the quality and speed of WB-MRI scans, enabling radiologists to acquire more detailed images with multiple sequences and orientations in an analogous time span [4].

Currently, WB-MRI is increasingly being used to evaluate a wider range of patients with different types of cancer [5,6]. For example, clinical guidelines from the European Society for Medical Oncology and the American Society of Clinical Oncology support the use of WB-MRI for staging prostate cancer, particularly in high-risk patients with inconclusive conventional imaging [7,8]. The International Myeloma Working Group (IMWG) guidelines advocate for the use of WB-MRI to identify bone lesions in patients suspected of having multiple myeloma or smoldering myeloma, especially those with prior negative or doubtful positron emission tomography–computed tomography (PET-CT) or low-dose WB-CT results, and as the initial imaging technique for patients with possible solitary plasmacytoma [9]. Additionally, WB-MRI is emerging as a valuable tool for cancer screening, both in individuals with genetic predispositions to cancer, supported by clinical guidelines [10,11,12], and in the general population [13].

In recent years, there has been a widespread adoption of standardized reporting systems, known as Reporting and Data Systems (RADSs), in radiology [14,15,16]. These systems aim to minimize variability and ambiguous terminology in imaging reports, thereby improving image interpretation and outcome tracking. Building on this trend, since 2017, three RADSs have been developed for application to WB-MRI in different clinical settings [17]: METastasis Reporting and Data System for Prostate Cancer (MET-RADS-P) [18] for men with advanced prostate cancer, Myeloma Response Assessment and Diagnosis System (MY-RADS) [19] for patients with multiple myeloma, and Oncologically Relevant Findings Reporting and Data System (ONCO-RADS) [20] for cancer screening.

The MET-RADS-P and MY-RADS guidelines (published in 2017 and 2019, respectively) demonstrate a high degree of overlap in their recommended imaging protocols. A “core” protocol, suggested in both guidelines for initial disease evaluation, requires approximately 30 min to complete. This streamlined approach provides sufficient coverage for the detection of bone marrow and extramedullary disease in patients with multiple myeloma, while also enabling effective evaluation of bone metastases and soft tissue involvement in patients with metastatic prostate cancer. Both sets of guidelines also include more extensive imaging protocols, referred to as “comprehensive assessment”. These expanded protocols are recommended for better evaluating soft tissue and visceral involvement in advanced prostate cancer or extramedullary disease in multiple myeloma. Moreover, the “comprehensive assessment” is particularly valuable for patients undergoing serial tumor response evaluation, including those participating in clinical trials [18,19]. The 2021 ONCO-RADS guidelines outline a “standard protocol” for WB-MRI in individuals with cancer predisposition syndromes. These guidelines also include a “short protocol” which may be more suitable for cancer screening in asymptomatic individuals within the general population [20].

Table 1 provides a summary and comparison of the imaging protocols used in the three WB-MRI RADSs [6].

The aim of this narrative review is to provide a summary of the scientific evidence that has emerged so far regarding the clinical utility of WB-MRI RADSs.

## 2. Materials and Methods

We conducted a comprehensive literature review using three major databases: Scopus, Web of Science, and PubMed. The search was up to date as of 4 December 2024. We searched for studies using keywords related to the three WB-MRI RADS guidelines: MET-RADS OR “METastasis Reporting and Data System” OR MY-RADS OR “Myeloma Response Assessment and Diagnosis System” OR ONCO-RADS OR “Oncologically Relevant Findings Reporting and Data System”. To maintain clarity and prioritize the most current and rigorous research, we restricted our search to original research articles published in English. Reviews, letters to the editor, and case reports were not included. Following an initial screening of titles and abstracts, 20 of the 40 articles were deemed relevant to our research aims. A full-text review of these articles was then undertaken to gain a comprehensive understanding of the existing literature.

## 3. MET-RADS-P

MET-RADS-P was developed to stage and monitor men with advanced prostate cancer with WB-MRI [18]. Prostate-specific antigen (PSA) has long been a cornerstone in monitoring prostate cancer progression [21], considering the limitations of conventional imaging (e.g., CT and bone scintigraphy), particularly in assessing sclerotic bone metastases [22]. Nevertheless, disease progression can occur in advanced prostate cancer without a corresponding rise in PSA levels [23]. In contrast, functional imaging modalities, such as WB-MRI with diffusion-weighted imaging (DWI), offer a more accurate assessment of disease progression and spread. This discrepancy between PSA levels and WB-MRI findings supports the clonal evolution theory of castration resistance, which suggests that prostate cancer consists of two distinct cell populations: androgen-dependent and androgen-independent cells. Hormonal therapy primarily targets androgen-dependent prostate cancer cells, which express PSA, leading to the outgrowth of more aggressive, androgen-independent tumor cells [24].

MET-RADS-P assesses disease burden in 14 specific body regions, including the primary tumor site, bone, lymph nodes, lung, liver, and other soft tissues. The evaluation is based on the morphological and signal appearance of disease on WB-MRI at baseline and follow-up. All DW images with apparent diffusion coefficient (ADC) values should be evaluated alongside corresponding anatomical and relative fat-fraction percentage (FF%) images. Generally, normal bone marrow ADC values are less than 600–700 μm^2^/s. Viable tumor tissue, on the other hand, typically exhibits ADC values ranging from 700 to 1400 μm^2^/s. ADC values greater than 1400 μm^2^/s are commonly observed in treated or necrotic disease [18]. FF% decreases with malignant infiltration and increases as cancer tissue is replaced by fatty bone marrow [25]. A qualitative evaluation of response, using a 5-point Response Assessment Category (RAC) scale (1: highly likely response, 2: likely response, 3: stable disease, 4: likely progression, 5: highly likely progression), is assigned to each anatomical region by comparing the current WB-MRI with the baseline or nadir examination. Moreover, MET-RADS-P can document mixed (discordant) responses using a three-pattern scoring system that assesses the primary, secondary, and tertiary RACs. The primary/dominant pattern represents the most common RAC observed within a region. The secondary pattern records the second most frequent RAC within the region. The tertiary pattern should only be used to document progressive disease (RACs 4 and 5) occurring in a minority of lesions within a region, if not already captured by the primary or secondary assessments [18].

### 3.1. Prognostic Role of MET-RADS-P

Confirming the prognostic value of the MET-RADS-P is essential, as this scoring system aims to provide a comprehensive assessment of advanced prostate cancer, which can inform treatment decisions.

Yoshida et al. retrospectively examined 73 patients with castration-resistant prostate cancer who underwent WB-MRI scans with DWI to assess disease progression before initiating further lines of treatment. The median number of prior hormonal therapies administered was four, with a range of one to ten. In total, 35% of the cohort had received prior taxane-based chemotherapy, while 42% had been previously treated with novel hormonal agents. Bone metastases were detected in the majority of patients (83%), while lymph node and visceral metastases were less common, occurring in 14% and 6% of patients, respectively. Patient survival varied significantly based on the extent of bone metastases and the presence of distant organ involvement, as assessed by MET-RADS-P scores. Multivariate analysis confirmed that a higher degree of bone metastases and the presence of visceral metastases were associated with poorer cancer-specific survival. Furthermore, in a model incorporating clinical variables and MET-RADS-P scores, the presence of visceral metastases and a history of multiple prior hormonal therapies were independently associated with significantly shorter cancer-specific survival [26]. In a separate study of 66 men with castration-resistant prostate cancer, Yamamoto et al. conducted a retrospective analysis of WB-DWI scans using ADC values. The median number of prior treatment regimens was three, ranging from one to eight. In particular, 26% of patients had received prior taxane-based chemotherapy, while 33% of patients had been previously treated with novel hormonal agents. The extent of metastatic disease, as measured by diffusion volume, was strongly associated with the MET-RADS-P score and was an independent predictor of poorer patient survival. When the extent of metastatic disease, as measured by diffusion volume with low ADC values (0.4–0.9 × 10^−3^ mm^2^/s), was included in the analysis, it emerged as a significant predictor of shorter survival, along with the number of prior treatments [27]. In a recent study, Van Damme et al. prospectively compared the ability of PSA levels and WB-MRI (according to RACs of MET-RADS-P) to predict response to therapy in 51 metastatic castration-resistant and 37 metastatic hormone-naïve prostate cancer men treated with androgen deprivation therapy and an androgen receptor pathway inhibitor. The agreement between PSA and WB-MRI response assessment was slight in the castration-resistant prostate cancer group (k: 0.15) and was fair in metastatic hormone-naïve prostate cancer (k: 0.30). In metastatic hormone-naïve prostate cancer, patients who responded to treatment, as assessed by either PSA levels or MET-RADS-P, were less likely to require additional therapies. Notably, the progression of disease detected by WB-MRI was significantly associated with a higher risk of death. In metastatic castration-resistant prostate cancer, the majority of patients with a decline in PSA levels had evidence of disease progression according to MET-RADS-P. Moreover, neither WB-MRI nor PSA predicted survival or the need for additional therapies [28].

### 3.2. Reliability of MET-RADS-P

MET-RADS-P guidelines aim to standardize the evaluation of metastatic disease response via WB-MRI in advanced prostate cancer. To ensure reliability, it is essential that these RADS undergo inter-observer agreement studies. Pricolo et al. retrospectively analyzed 50 WB-MRI exams from 31 patients with advanced prostate cancer. They assessed the agreement between an experienced radiologist and a radiology resident who applied MET-RADS-P to evaluate metastases detection and primary and secondary RACs. Both the readers identified a comparable number of metastatic sites overall. The inter-reader agreement for the primary RACs was excellent in assessing the spine, limbs, pelvis, lungs, and other sites (k: 0.81–1.0), substantial in evaluating the liver, thorax, retroperitoneal nodes, and other nodes (k: 0.61–0.80), and moderate for pelvic nodes (k: 0.56). For the secondary RACs, the inter-observer agreement was almost perfect in assessing retroperitoneal nodes and cervical spine (k: 0.89–0.93), substantial for the evaluation of thorax, dorsal spine, limbs, pelvis, and pelvic nodes (k: 0.61–0.80), and moderate for the lumbosacral spine (k: 0.44). The overall agreement regarding the final patient management was almost perfect (k: 0.92) [29]. Subsequently, Liu et al. evaluated the consistency among radiologists using a structured reporting tool based on MET-RADS-P guidelines focusing on pelvic MRIs. Two experienced and two young radiologists retrospectively analyzed 163 pelvic MRIs from 105 patients with advanced prostate cancer, classifying metastatic lesion detection according to primary and secondary RACs, both with and without a structured reporting tool. All radiologists demonstrated higher agreement with the reference standard for metastasis detection when using the structured report compared to the conventional report, with better performances of senior radiologists compared to young radiologists (maximum k: 0.83 vs. 0.66). Furthermore, the inter-observer agreement using structured reports between the two experienced radiologists was almost perfect for primary RACs and substantial for secondary RACs (k: 0.81 and 0.75, respectively), while the agreement between the two junior readers was substantial for both primary and secondary RACs (k: 0.76 and 0.68, respectively) [30]. In another study by Liu et al., the consistency between two radiologists in assessing the response to treatment in nodes of 162 prostate cancer patients using pelvic MRI and MET-RADS-P was almost perfect for target lesions, non-target lesions, and non-pathological lesions (k: 0.90, 0.95, and 0.84, respectively). Moreover, the authors implemented a semi-automated technique utilizing deep learning-based lymph node segmentation that accurately evaluated all target, non-target, and non-pathological lesions, adhering to the MET-RADS-P criteria and demonstrating high agreement with the assessments of radiologists. These preliminary findings suggest a promising approach toward developing a fully automated algorithm for assessing treatment response based on the MET-RADS-P guidelines [31]. Agazzi et al. assessed the agreement between two readers in evaluating treatment response using FF% and MET-RADS-P criteria in 34 hormone-naive prostate cancer patients with bone metastases. They observed moderate agreement for small lesions [<1 cm, intraclass correlation coefficient (ICC): 0.64]. However, higher agreement was found for lesions >1 cm, particularly when evaluating volumetric measurements on multiple slices (ICC: 0.88) [32].

**Figure 1:**
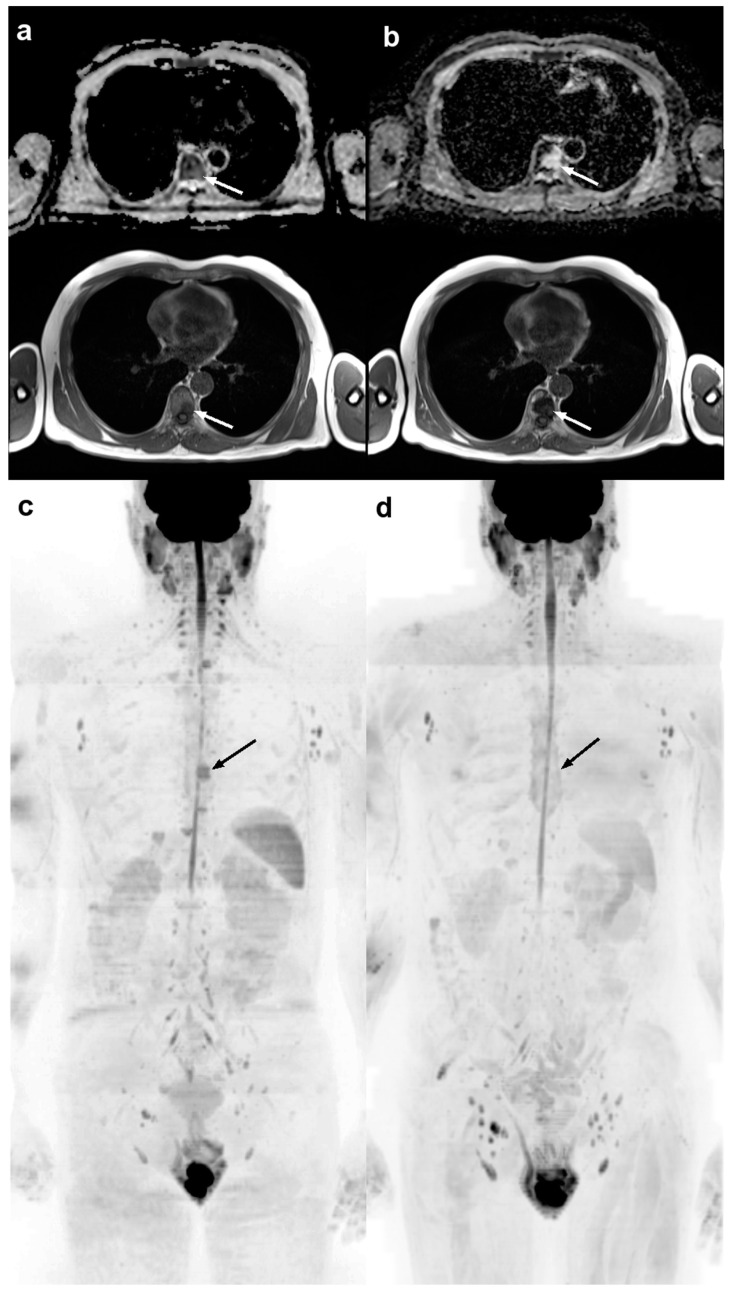
Whole-body magnetic resonance images of a 67-year-old man with metastatic hormone-sensitive prostate cancer showing response to treatment, with primary/dominant Response Assessment Category (RAC) of 1. (**a**) Axial apparent diffusion coefficient (ADC) map (**upper**) and T1-weighted (**lower**) images at the start of luteinizing hormone releasing hormone agonist therapy show the presence of a dorsal spine lesion (arrows) with ADC value of 784 μm^2^/s. (**b**) Axial ADC map (**upper**) and T1-weighted (**lower**) images at the follow-up show an increase in the spine lesion size accompanied by an increase in the ADC value (1608 μm^2^/s), suggestive for edema. Thus, this picture was classified as indicative of a highly likely response. Three-dimensional b900 maximum intensity projection images (**c**) at start of therapy and (**d**) at follow-up confirm the disappearance of the spine lesion. Reprinted adapting the caption from Pricolo et al. [29] under the terms and conditions of the Creative Commons Attribution 4.0 International License.

Figure 1 shows an example of an evaluation of treatment response according to MET-RADS-P.

### 3.3. Summary

Initial studies have primarily focused on evaluating the prognostic role of MET-RADS-P and its reliability (Table 2).

MET-RADS-P emerged as a prognostic imaging biomarker for castration-resistant prostate cancer undergoing multiple lines of treatment, also correlated with quantitative assessments, and enhanced the information provided by PSA response assessments. Furthermore, MET-RADS-P correlated with both survival and time to subsequent hormonal therapy in metastatic hormone-sensitive prostate cancer. These results underscore the value of integrating the MET-RADS-P framework into future clinical trials, enhancing patient selection and facilitating the monitoring of responses to contemporary hormonal therapies.

MET-RADS-P showed high reliability in detecting metastases and in assessing treatment response in bone metastases, also between radiologists with different levels of experience. Some level of disagreement was observed in the assessment of the mixed response to treatment (secondary RACs), among small bone metastases and in extraosseous sites like pelvic lymph nodes.

Additional studies with larger cohorts, a prospective design, and multicenter participation, involving the assessments of more than two radiologists, are required to validate these encouraging preliminary findings.

The use of standardized reporting templates and artificial intelligence tools could enhance the consistency of MET-RADS-P and streamline the evaluation process in clinical settings. For instance, an artificial intelligence model was developed to automatically segment pelvic lymph nodes on MRI. By analyzing the quantitative measurements derived from this automated segmentation, it could be possible to assess treatment response using the MET-RADS-P criteria [31]. It is reasonable to expect that in the future, additional similar software will be developed, suitable for applications beyond lymph node assessment, such as bone evaluation.

## 4. MY-RADS

MY-RADS was developed to stage and monitor patients with multiple myeloma with WB-MRI [19]. WB-MRI, including DWI, has become established as the most sensitive technique for bone marrow imaging [33,34]. The correlation between ADC values and cellular density enables the early assessment of treatment response, preceding observable changes in lesion size. Furthermore, ADC measurements can facilitate the identification of heterogeneity in treatment response [35,36]. Skeletal surveys and low-dose WB-CT primarily assess bone destruction, limiting their sensitivity for detecting disease within the bone marrow and their utility as restaging tools [37,38]. While PET-CT can aid in the diagnosis of focal bone lesions, MRI is generally more sensitive [33,34].

MET-RADS-P guidelines served as a model for the development of MY-RADS. MY-RADS assesses disease burden in eight specific bone regions, including the spine, pelvis, long bones, skull, and ribs [19]. Additionally, it evaluates para/extramedullary [39] and diffuse disease. Multisequence evaluations should incorporate DWI with ADC maps, along with anatomic and FF% images. Similar to MET-RADS-P, a 5-point RAC scale is used to qualitatively assess response in each anatomical region by comparing current and baseline/nadir WB-MRI [19].

### 4.1. Prognostic Role of MY-RADS

Confirming the prognostic value of MY-RADS is crucial, as it provides a comprehensive assessment of multiple myeloma and guides treatment decisions.

In a cohort of 30 newly diagnosed multiple myeloma patients (of whom 16 had diffuse disease), MY-RADS criteria served as the reference standard for diagnosing diffuse infiltration. Patients with diffuse disease exhibited a mean bone marrow ADC of 667 μm^2^/s, significantly higher than the 489 μm^2^/s observed in patients without diffuse disease. Using an ADC threshold of 590 μm^2^/s, diffuse disease was detected with an area under the curve of 0.82, with a sensitivity of 0.81 and a specificity of 0.78. Moreover, a statistically significant difference was found in the percentage of bone marrow plasma cells among patients with varying degrees of diffuse infiltration [40]. Belotti et al. conducted a retrospective study to assess the prognostic significance of the MY-RADS RACs in 64 newly diagnosed multiple myeloma patients who underwent autologous stem cell transplantation. Patients who attained a complete imaging response (RAC 1) exhibited significantly improved progression-free survival and overall survival outcomes compared to patients with residual disease at imaging (RAC ≥ 2). Moreover, WB-DWI identified residual disease in 20% of patients who had undetectable levels of minimal residual disease as assessed by multiparametric flow cytometry. Multivariate analysis identified high-risk cytogenetics and WB-DWI persistent disease (RAC ≥ 2) as independent predictors of post-transplant progression-free survival [41]. In a prospective study, Agarwal et al. enrolled 55 patients with plasma cell dyscrasias. Baseline and 6-month WB-DWI and serum biomarker measurements were obtained for each patient. In both newly diagnosed and relapsed multiple myeloma, patients with at least one focal lesion identified on MRI had a higher percentage of plasma cells in their diagnostic bone marrow aspirate compared to those without detectable lesions at imaging. ADC values exhibited a moderate inverse correlation with serum B-cell maturation antigen levels, a well-established marker of increased tumor burden. Moreover, most IMWG responders were scored as RAC 1, while most non-responders were scored as RAC 3 [42]. Dong et al. retrospectively studied 56 newly diagnosed multiple myeloma patients with baseline WB-MRI. Only among patients without anemia, those who achieved a deep response to induction chemotherapy had significantly lower total burden scores as assessed by the MY-RADS criteria (cutoff ≤ 15), higher FF% (>16%), and lower ADC values (≤0.84 × 10^−3^ mm^2^/s), compared to those who did not achieve a deep response. The combination of these three parameters achieved a sensitivity of 94%, specificity of 93%, and accuracy of 94% for predicting deep response [43]. Paternain et al. retrospectively evaluated 27 multiple myeloma patients with baseline and post-treatment WB-MRI and PET-CT. The mean ADC of lesions showed a significant negative correlation with their corresponding maximum standardized uptake value (SUVmax). Post-treatment, responders had significantly higher mean ADC (1.58 × 10^−3^ mm^2^/s) and lower SUVmax (2.05) compared to non-responders (0.70 × 10^−3^ mm^2^/s and 5.33, respectively). Strong agreement was observed between IMWG criteria and both MY-RADS RACs (k: 0.85) and PET (k: 0.77) criteria. In particular, RAC criteria demonstrated high sensitivity (87%), specificity (100%), positive predictive value (100%), and negative predictive value (86%) [44]. Discordant results emerged from the prospective study by Mesguich et al. They enrolled 30 patients with newly diagnosed multiple myeloma. Each patient underwent PET-CT and WB-MRI at baseline, post-induction chemotherapy, and post-autologous stem cell transplantation. MY-RADS did not demonstrate an independent prognostic impact on progression-free survival. Nevertheless, a positive PET-CT scan, either after induction therapy or after autologous stem cell transplantation, was associated with reduced progression-free survival [45].

**Figure 2:**
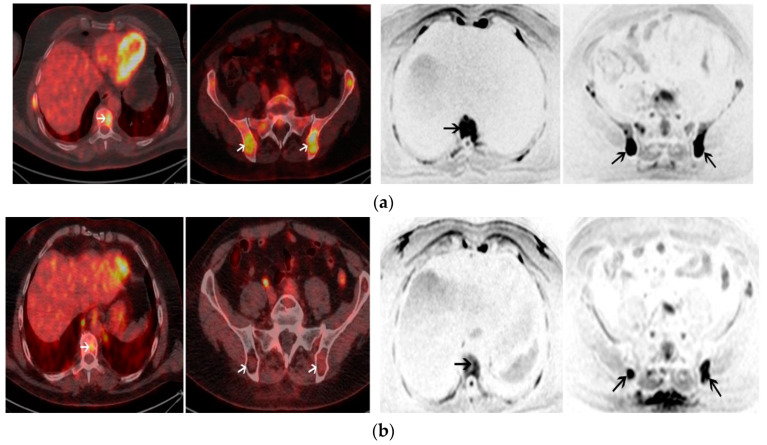
Positron emission tomography-computed tomography (PET-CT) and whole-body diffusion-weighted imaging (WB-DWI) of a 55-year-old male with IgA kappa multiple myeloma. (**a**) Axial images of baseline PET-CT (**left panel**) show multiple focal lesions with high uptake including one lesion involving T9 and two lesions involving the ilium (white arrows). Axial images of baseline WB-DWI (**right panel**) show lesions with restricted diffusion (b = 800 s/mm^2^) also involving T9 and the ilium with an apparent diffusion coefficient ADC of 920 μm^2^/s (black arrows). (**b**) Positive post-autologous stem cell transplantation (ASCT) PET-CT (**left panel**) shows a persistent uptake of T9 (maximum standardized uptake value > liver uptake) and the regression of uptakes within lesions involving the ilium. Positive post-ASCT WB-DWI (**right panel**) shows a regression of the restricted diffusion in T9 but the persistence of two lesions in the ilium with restricted diffusion and ≤25% increase in the ADC values (ADC = 1000 μm^2^/s). The subject was in complete remission after ASCT but relapsed 16 months after ASCT. Reprinted adapting the caption from Mesguich et al. [45] under the terms and conditions of the Creative Commons Attribution 4.0 International License.

Figure 2 shows an example of post-autologous stem cell transplantation response assessment on PET-CT and WB-MRI.

### 4.2. Reliability of MY-RADS

The MY-RADS guidelines were designed to enhance standardization and minimize variability in the acquisition and reporting of WB-MRI in multiple myeloma. Consequently, similar to MET-RADS-P, inter-observer agreement studies are crucial for validating this score. In 56 multiple myeloma patients, two radiologists showed excellent agreement (ICC: 0.95–0.99) in assigning MY-RADS total burden scores and measuring ADC and FF in diffuse and focal disease [43]. In a retrospective analysis by Rossi et al., the intra-reader and inter-reader agreement for assigning the MY-RADS score in 54 multiple myeloma patients was considered good. The intra-reader agreement for two radiologists was substantial (k: 0.78 and k: 0.76, respectively), as was the inter-reader agreement (k: 0.65) [46]. In a prospective study, Wennmann et al. assessed 140 focal bone marrow lesions in 37 patients with monoclonal plasma cell disorder. Each patient underwent two identical WB-MRI scans according to the MY-RADS protocol with patient repositioning between scans. A variation of 1–2 mm in the measurements of focal bone marrow lesions was observed in the majority of cases, both on repeat measurements by the same radiologist and on measurements performed by different radiologists, without any underlying biological change in the lesion [47].

To broaden the clinical availability of WB-MRI beyond specialist centers, consistent high-quality imaging must be achievable across diverse hardware and software platforms. Thus, multicenter imaging studies are indispensable for facilitating the transition of quantitative biomarkers (e.g., ADC and FF%) from research to clinical practice [48]. WB-MRI according to the MY-RADS protocol was successfully deployed at 12 sites, utilizing three distinct vendor systems and two field strengths. Four primary imaging protocols were established, with protocol modifications required for four sites to address hardware and software limitations. Nevertheless, the adoption of standardized methodology and imaging protocols facilitated the acquisition of images suitable for both qualitative and quantitative analysis across all participating sites [49]. In a multicenter study involving ten sites with varying levels of WB-MRI experience and utilizing three different scanner manufacturers and two field strengths, a qualitative radiological review of 121 WB-MRIs demonstrated that 94% of examinations were rated as having good or excellent image quality. Only one examination was considered non-diagnostic. Low-quality DWI, characterized by poor signal-to-noise ratio, anterior thoracic signal loss, and brain geometric distortion, was the primary factor contributing to overall poor examination quality [50].

### 4.3. Summary

Preliminary investigations have mainly focused on evaluating the prognostic value and reliability of MY-RADS (Table 3).

The majority of studies have demonstrated that MY-RADS is a prognostic imaging biomarker for multiple myeloma, correlating with quantitative imaging assessments and providing complementary information to that offered by serum biomarkers. Additionally, MY-RADS correlated with survival and predicted treatment response, aligning with IMWG response groups. These findings highlight the importance of integrating MY-RADS into future clinical trials to optimize patient selection and treatment monitoring strategies.

While the evidence regarding inter-reader agreement of MY-RADS is currently promising, it remains limited. On the other hand, multicenter studies have validated the use of MY-RADS in various clinical settings and with different equipment and software.

Additional studies with larger patient samples, a prospective design, and the assessments of multiple radiologists with varying levels of experience are required to definitively validate these encouraging preliminary findings. Furthermore, it would be desirable to validate artificial intelligence software that could assist in the use of MY-RADS and further promote the dissemination of this score.

## 5. ONCO-RADS

ONCO-RADS was developed to standardize the utilization of WB-MRI for cancer screening in individuals with cancer predisposition syndromes, with the long-term goal of extending its application to the general population [20].

Annual WB-MRI, along with contrast-enhanced brain MRI (and breast MRI for adult women), is the recommended imaging strategy for individuals with Li–Fraumeni syndrome [10]. Annual WB-MRI is advised for patients with constitutional mismatch repair deficiency syndrome from age 6 and for those with hereditary retinoblastoma from age 8 [11,51]. WB-MRI screening is recommended for individuals with hereditary paraganglioma and pheochromocytoma syndromes [12]. Moreover, there is growing interest in using whole-body MRI as a screening modality for asymptomatic individuals in the general population to detect early-stage cancers [13].

ONCO-RADS was formulated by adapting the frameworks of MET-RADS-P and MY-RADS. Multisequence evaluations, including DWI with ADC maps, T1-weighted images, T2-weighted images, and FF% images, should be performed. A systematic approach should be used to classify all abnormal findings into one of the following seven anatomic districts: head, neck, chest, abdomen, pelvis, bones, and limbs. For each abnormal finding, a detailed description should be provided, along with an assigned ONCO-RADS category (1–5). This categorization system estimates the likelihood of the finding being oncologically relevant, with 1 indicating a normal finding and 5 indicating a highly likely malignant finding. Asymptomatic individuals with normal (ONCO-RADS 1) or benign (ONCO-RADS 2) findings typically require no further follow-up. Individuals in high-risk groups with ONCO-RADS 1–2 findings should undergo repeat whole-body MRI as recommended by specific guidelines. Asymptomatic individuals with ONCO-RADS 3 findings are considered to have an intermediate risk of cancer. Individuals with ONCO-RADS 3–5 findings from high-risk groups or ONCO-RADS 4–5 findings from the general population should undergo further evaluation, potentially including biopsy [20].

The scientific evidence supporting ONCO-RADS remains quite limited at this time and requires further studies. This is likely due to its relatively recent introduction compared to established systems like MET-RADS-P and MY-RADS, as well as the limited widespread use of WB-MRI for cancer screening. ONCO-RADS has demonstrated potential as a valuable tool for cancer screening in the general population. Moreover, its implementation has not been associated with negative impacts on quality of life, even considering the increased detection of incidental findings both in the general population and in high-risk groups.

Hu et al. retrospectively analyzed 2064 WB-MRI from asymptomatic individuals who underwent cancer screening. Two blinded radiologists classified findings using ONCO-RADS. The interobserver reliability for the ONCO-RADS assignment was nearly perfect (k: 0.93). A small percentage of participants (2.1%) had findings categorized as ONCO-RADS 4 or 5, indicating a high likelihood of malignancy, and 1.2% were diagnosed with malignancy. Cancer prevalence increased with ONCO-RADS category: 0.1% (2), 5.4% (3), 42.9% (4), and 75% (5). In the multivariate analysis, previous surgery, hepatitis B carrier status, hypertension, and older age were independently associated with findings categorized as ONCO-RADS 4 or 5 [52]. Neves et al. aimed to assess the feasibility of a WB-MRI screening protocol for children and young adults with ataxia telangiectasia. Their study demonstrated high patient adherence, with 83% of participants completing the protocol. A high percentage (93%) of completed WB-MRI scans achieved adequate image quality. Two patients with ONCO-RADS 3 findings required further investigation. WB-MRI for cancer screening in A-T is technically feasible and well tolerated by the children and young people undergoing the scan. Furthermore, while families generally had a positive view of WB-MRI cancer screening, they also emphasized the importance of clear communication, practical support, and addressing short-term anxiety [53]. Conti et al. studied the long-term psychological impact of unexpected abnormal findings detected by WB-MRI cancer screening in 121 asymptomatic individuals. All 121 participants were found to have abnormal imaging results. Of these, 101 were classified as ONCO-RADS 2 (benign) and 19 as ONCO-RADS 3 (indeterminate). Overall, the participants exhibited only a minor increase in depressive symptoms after one year. The severity and quantity of abnormal findings were not significantly associated with changes in psychological well-being. Changes in depressive and anxious symptoms over time were significantly associated with the personality traits of openness and conscientiousness [54].

**Figure 3:**
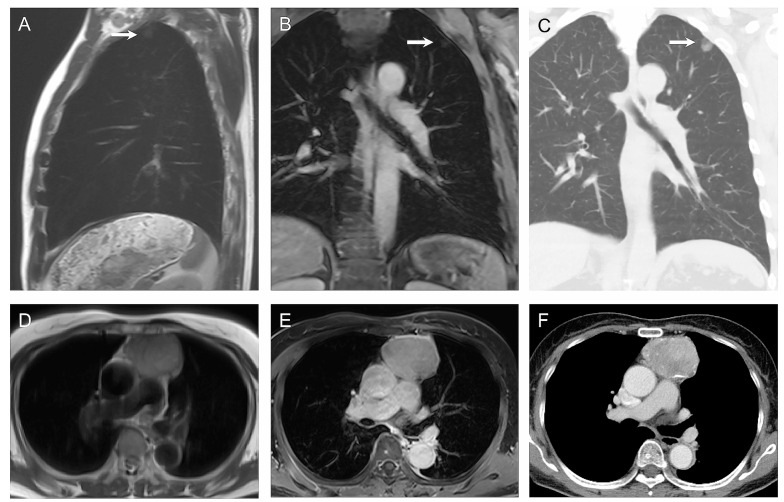
Imaging findings with Oncologically Relevant Findings Reporting and Data System category 4 in the chest region of two different patients. Sagittal T2-weighted half-Fourier single-shot turbo spin echo (HASTE) (**A**) and coronal contrast-enhanced T1-weighted gradient echo (GRE) (**B**) images reveal 17 mm subpleural nodule (arrows) in upper lobe of the left lung in a 62-year-old man. The lesion is seen on the corresponding coronal computed tomography (CT) scan lung window (**C**) and was confirmed as lung adenocarcinoma after surgical resection. In a 69-year-old woman, a 5.8 cm anterior mediastinal mass with hyperintensity was noted on the T2-weighted HASTE image (**D**) and contrast enhancement on the T1-weighted GRE image (**E**). The corresponding axial CT scan (**F**) demonstrates calcification in mass and thymoma was diagnosed after surgical resection. Reprinted adapting the caption from Hu et al. [52] under the terms and conditions of the Creative Commons Attribution 4.0 International License.

Figure 3 shows examples of ONCO-RADS assignments in the general population for cancer screening.

Table 4 summarizes the main studies validating the ONCO-RADS.

## 6. Conclusions

While preliminary studies have yielded promising and mostly concordant results for the prognostic value and reliability of WB-MRI RADSs, there is a need for more research, especially regarding ONCO-RADS and inter-reader agreement for MY-RADS. Additional large-scale, prospective, multicenter studies are required to confirm and expand upon these encouraging initial findings.

## Figures and Tables

**Table 1:** Summary of whole-body magnetic resonance imaging reporting and data systems protocols. ADC, apparent diffusion coefficient. DWI, diffusion-weighted imaging. FLAIR, fluid-attenuated inversion recovery. GRE, gradient echo. MET-RADS-P, METastasis Reporting and Data System for Prostate Cancer. MIP, maximum intensity projection. MY-RADS, Myeloma Response Assessment and Diagnosis System. ONCO-RADS, Oncologically Relevant Findings Reporting and Data System. STIR, short inversion time inversion recovery. T1-w, T1-weighted; T2-w, T2-weighted. TSE, turbo spin echo. VIBE, volumetric interpolated breath-hold examination.

	MET-RADS-P for Advanced Prostate Cancer	MY-RADS for Multiple Myeloma	ONCO-RADS for Cancer Screening
Whole-body field of view	Vertex to mid-thighs	Vertex to knees	Vertex to feet (vertex to mid-thighs in short protocol)
Whole-body axial T1-w GRE Dixon with fat and water image reconstructions to generate fat fraction maps	Yes (axial and coronal for comprehensive assessment)	Yes (axial and coronal for comprehensive assessment)	Yes
Whole-body axial DWI with ADC values calculation and coronal rotational MIP of high b value images	Yes (supplementary b value for comprehensive assessment)	Yes (supplementary b value for comprehensive assessment)	Yes
Whole-body axial T2-w TSE	Optional (required for comprehensive assessment)	Optional (required for comprehensive assessment)	Yes
Spine sagittal T1-w TSE	Yes	Yes	Yes (not required for short protocol)
Spine sagittal T2-w STIR	Yes	Yes	Yes
Brain axial T2-w FLAIR	Optional for comprehensive assessment	Optional for comprehensive assessment	Yes
Lung axial T1-w GRE VIBE	Optional for comprehensive assessment	Optional for comprehensive assessment	Yes
Regional assessments	Only for comprehensive assessment (e.g., dedicated prostate, brain, and spine studies, also with contrast agent injection)	Optional for comprehensive assessment (e.g., symptomatic or known sites outside standard field of view, sites of spinal cord or nerve root compression, extramedullary disease)	Yes, in specific cancer predisposition syndrome (e.g., dedicated brain, upper and lower limbs imaging, also with contrast agent injection)

**Table 2:** Summary of main studies evaluating the role of METastasis Reporting and Data System for Prostate Cancer (MET-RADS-P), listed in order of citation in the text. MRI, magnetic resonance imaging.

Investigators	Study Design	Conclusions
Yoshida et al. [26]	Retrospective	Bone metastatic volume and visceral metastasis, assessed by MET-RADS-P, were prognostic factors for overall survival in castration-resistant prostate cancer patients
Yamamoto et al. [27]	Retrospective	Metastasis diffusion volume correlated with MET-RADS-P scores and was a prognostic factor for castration-resistant prostate cancer patients, especially when based on apparent diffusion coefficient values
Van Damme et al. [28]	Prospective	Progression of metastatic disease according to MET-RADS-P in hormone-naïve prostate cancer patients was associated with an increased risk of subsequent therapeutic intervention and reduced overall survival.
Pricolo et al. [29]	Retrospective	Inter-observer agreement for MET-RADS-P was excellent for bone assessment, regardless of reader expertise, but varied for other body regions
Liu et al. [30]	Retrospective	Radiologists with different levels of expertise demonstrated high inter-reader agreement using a structured reporting system based on MET-RADS-P guidelines for pelvic MRI
Liu et al. [31]	Retrospective	The accuracy of response assessments of automatically segmented lymph nodes closely approximated that of manually segmented lymph nodes
Agazzi et al. [32]	Prospective	Fat fraction percentage assessment was highly reproducible for bone metastases > 10 mm

**Table 3:** Summary of main studies evaluating the role of Myeloma Response Assessment and Diagnosis System (MY-RADS), listed in order of citation in the text. ADC, apparent diffusion coefficient. IMWG, International Myeloma Working Group. PET-CT, positron emission tomography-computed tomography. RAC, Response Assessment Category. WB-DWI, whole-body diffusion-weighted imaging. WB-MRI, whole-body magnetic resonance imaging.

Investigators	Study Design	Conclusions
Mesguich et al. [40]	Prospective	Using MY-RADS criteria, an optimal ADC cutoff of 590 μm^2^/s was identified for diagnosing diffuse disease
Belotti et al. [41]	Retrospective	MY-RADS RACs independently stratified multiple myeloma patients and predicted prognosis after autologous stem cell transplantation. Combining WB-DWI with multiparametric flow cytometry enhanced minimal residual disease assessment
Agarwal et al. [42]	Prospective	MY-RADS and serum biomarkers could assess tumor burden and bone loss in plasma cell dyscrasias
Dong et al. [43]	Retrospective	In newly diagnosed multiple myeloma patients without anemia, a low MY-RADS total burden score, low ADC, and high fat fraction predicted early treatment response
Paternain et al. [44]	Retrospective	WB-DWI and ADC values according to MY-RADS could assess multiple myeloma treatment response, correlating well with IMWG and PET-CT criteria
Mesguich et al. [45]	Prospective	PET-CT, whether post-induction or post-autologous stem cell transplantation, was a stronger prognostic biomarker than MY-RADS for newly diagnosed multiple myeloma patients
Rossi et al. [46]	Retrospective	Good intra- and inter-reader agreement following MY-RADS guidelines
Wennmann et al. [47]	Prospective	Small variations in focal bone marrow lesion size were common, likely due to patient positioning or inter-rater variability. Understanding this measurement uncertainty is crucial when interpreting MY-RADS-based changes in lesion size
Rata et al. [49]	Prospective	The implementation and support of standardized MY-RADS WB-MRI protocols in prospective multicenter clinical trials could be feasible, even at sites with limited prior experience in WB-MRI
Keaveney et al. [50]	Prospective	Multicenter WB-MRI studies employing the MY-RADS protocol could be successfully implemented, regardless of differences in MRI hardware or prior WB-MRI expertise among participating sites

**Table 4:** Summary of main studies evaluating the role of Oncologically Relevant Findings Reporting and Data System (ONCO-RADS), listed in order of citation in the text. WB-MRI, whole-body magnetic resonance imaging.

Investigators	Study Design	Conclusions
Hu et al. [52]	Retrospective	ONCO-RADS was validated in the general population and associated with cancer risk. Older age, hypertension, hepatitis B, and prior surgery were risk factors for high-risk findings (ONCO-RADS ≥ 4)
Neves et al. [53]	Prospective	WB-MRI was a feasible and well-tolerated screening tool for children and young adults with ataxia telangiectasia and their families
Conti et al. [54]	Prospective	WB-MRI according to ONCO-RADS could be used in asymptomatic individuals for cancer screening without causing long-term psychological harm. Certain personality traits could exacerbate psychological distress within 1 year after WB-MRI in individuals with abnormal findings
